# HIV prevalence in South Africa through gender and racial lenses: results from the 2012 population-based national household survey

**DOI:** 10.1186/s12939-019-1055-6

**Published:** 2019-10-30

**Authors:** M. Mabaso, L. Makola, I. Naidoo, L. L. Mlangeni, S. Jooste, L. Simbayi

**Affiliations:** 1 Social Aspects of Public Health Research Programme, Humans Sciences Research Council, Durban, South Africa; 2 Department of Psychology, University of KwaZulu-Natal, 238 Mazisi Kunene Road, Glenwood, Durban, 4041 South Africa; 3 Social Aspects of Public Health Research Programme, Humans Sciences Research Council, Cape Town, South Africa; 4 Office of the Deputy Chief Executive Officer for Research, Human Sciences Research Council, Cape Town, South Africa

**Keywords:** Gender and racial disparities, HIV prevalence, South Africa

## Abstract

**Background:**

In South Africa, persistence of the HIV epidemic and associated gender and racial disparities is a major concern after more than 20 years of democratic dispensation and efforts to create a more healthy and equal society. This paper profiles HIV prevalence and related factors among Black African men and women compared to other race groups in South Africa using the 2012 population-based national household HIV survey.

**Methods:**

This secondary data analysis was based on the 2012 population-based nationally representative multi-stage stratified cluster random household sample. Bivariate and multiple logistic regression analysis were used to assess the relationship between HIV prevalence and associated factors by gender and racial profile.

**Results:**

Overall HIV prevalence was significantly higher (*p* < 0.001) among both Black African males (16.6%; 95% CI: 15.0–18.4) and females (24.1%; 95% CI: 22.4–26.0) compared to their counterparts from other races. Among Black African males, increased risk of HIV was significantly associated with age group 25–49 years and those 50 years and older compared with young males 15–25 years. Among all males, reported condom use at last sex was significantly associated with increased risk of HIV. High socio-economic status (SES) and perceived risk of HIV were associated with a decreased risk of HIV. Among female condom use at last sex and ever testing for HIV was associated with increased prevalence of HIV only among Black African females. Lower prevalence of HIV was associated with marriage, tertiary education, high SES, having a partner five years younger, perceived risk of HIV, and awareness of HIV status among Black African females.

**Conclusion:**

Gender and racial disparities rooted in structural and contextual inequalities remain important factors for the maintenance of the generalized HIV epidemic in the country. HIV prevention interventions need to cut across all strata of society but also target risk factors salient for specific groups. Alleviating vulnerability to HIV along gender and racial lines should also be viewed as part of a broader public health strategy.

## Background

Globally, both gender and racial inequalities play significant roles in perpetuating the HIV epidemic [1]. South Africa carries the largest share of the global HIV burden and a nationally representative population based household survey conducted in 2012 showed that an estimated 6.4 million people, about 12.2% of the population, were living with HIV in the country (2). The epidemic disproportionately affects women compared to their male counterparts, and is highest among Black African females [2–4]. The gender dynamics of HIV infection can be traced back to the first national HIV survey in 2002 showing differential infection rates by gender with significantly higher prevalence among females (17.7%) than males (12.8%) [5]. This trend showing gender differences has been consistently found in subsequent surveys. In the 2005 national survey a higher HIV prevalence was also recorded among females (13.3%) than males (8.2%) [6]. Similarly, in the 2008 national survey HIV prevalence was higher among females (17.3%) than males (11.6%) [7]. The picture was similar in 2012 with a higher HIV prevalence among females (14.4%) than males (9.9%) [2].

In all four national HIV surveys, Black Africans and especially women had the highest overall HIV prevalence compared to other race groups [2, 8–11]. Poverty is an overarching factor that increases the disparity associated with HIV prevalence between genders and among race groups created by historical and current unequal cultural, social and economic status in South Africa [12–15]. The low socio-economic status of women reinforce unequal gender power dynamics, which forces them into relationships that expose them to a higher risk of HIV infection than men, by engaging in risky sexual behaviours such as transactional and intergenerational sex [13–16]. Even in other sub-regions of east, west and central Africa, the population most affected by HIV are women and especially young women. This has been attributed to their unequal cultural, social and economic status in these societies [16, 17].

In South Africa, evidence shows that in addition to the gendered nature of the HIV epidemic, there are historical social conditions, which perpetuated race-based inequalities. These conditions increased susceptibility of certain groups to HIV infections, especially Black Africans who represent the majority of the population [8]. Racially perpetuated HIV is rooted on the socio-political economic system from the apartheid era in South Africa, which perpetuated an inherently unequal society [9]. This affected access to quality education, health and employment opportunities including breakdown of family structural norms due to migrant labour which in turn perpetuated the transmission of HIV [2, 9–11]. Similarly, In the United States of America studies observed that racial disparity of HIV is influenced by social and economic inequities linked to poverty, social exclusion and growing disparity in healthcare, which places many African American communities, although a racial minority in that country at high risk of HIV acquisition [17–21].

In South Africa, apartheid policies were focused on economic and health advances for the minority White race group in the first 80 years of the twentieth century [22]. Consequently, the Black majority race group living in South Africa suffered extreme inequalities simply by virtue of their race with the burden of HIV in particular reaching the highest epidemic proportions [23]. Persistence of the HIV epidemic in the context of historically entrenched disparities remains a major concern more than 20 years into the democratic dispensation. Attention has been paid to understanding the gender norms, stereotypes and practices, which contribute to the gendered nature of the HIV, such as male-female roles in sexual relationships. However, the racial dimension has not been fully considered as part of the gender lenses for HIV in the complex South African context. Although there are major efforts at redress in South Africa, Black African communities remain the most marginalised in all societal echelons. Black African women in particular are the most vulnerable than any other group, to the social, health and economic burden of HIV [2, 5–7].

Consequently, the concept of race and an unequal society still bears relevance in South African society and the Human Sciences Research Council (HSRC) bio-behavioural cross sectional HIV surveys have collected data for race in all survey waves. This paper investigates factors associated with HIV prevalence among Black African men and women compared to other race groups in South Africa using the 2012 population-based national household survey.

## Methods

### Study data

The study used 2012 data from the South African national population-based household survey conducted by the HSRC. The sampling was based on a multistage stratified cross-sectional design described in detail elsewhere [2]. Enumeration areas (EAs) selected from the 2001 population census and mapped by aerial photography to create the master sample that informed the sampling of households [24]. A systematic probability sample of 15 households was drawn from each of the 1000 randomly selected EAs from 86,000 EAs. The selection of EAs was stratified by province and four locality types were defined as urban formal, urban informal, rural formal (including commercial farms) and rural informal localities (including tribal authority areas). Persons of all ages living in the selected households were eligible to participate in the survey.

Household and age-appropriate individual questionnaires were verbally administered to consenting eligible individuals to solicit information on demographic characteristics, HIV related knowledge, attitudes, and behaviours and health issues [2]. Dried blood spot (DBS) specimens were also collected from consenting individuals using a finger prick. Samples were tested for HIV at accredited laboratories using an enzyme immunoassay (EIA) (Vironostika HIV Uni-Form II plus O, Biomeriux, Boxtel, The Netherlands), and samples which tested positive were retested using a second EIA (Advia Centaur XP, Siemens Medical Solutions Diagnostics, Tarrytown, NJ, USA). Any samples with discordant results on the first two EIAs were tested with a third EIA (Roche Elecys 2010 HIV Combi, Roche Diagnostics, Mannheim, Germany). The current study focused on those 15 years and older who agreed to participate in the study.

### Ethical consideration

The data were anonymised to ensure confidentiality. All persons who agreed to participate in the survey were required to provide either written or verbal consent for both the interview and specimen collection. Verbal consent was sought from participants who could not write or read. The Centre for Disease Control and Prevention (CDC) granted a waiver of written consent per 45CFR46 for respondents who were unable to provide written consent but consented verbally. Field staff signed their name on behalf of the respondent to certify the respondent had given that informed consent verbally. Additionally the field supervisor signed as a witness to certify that the respondent was fully aware of the consent procedure. Ethical approval for the study was obtained from the Research Ethics Committee of the Human Sciences Research Council, South Africa (REC: 5/17/11/10) as well as by the Associate Director of Science of the National Centre for HIV and AIDS, Viral Hepatitis, STD and TB Prevention at the USA’s (CDC) in Atlanta, Georgia, USA. The dataset(s) are available through the Human Sciences Research Council data research repository via the following link: http://curation.hsrc.ac.za/doi-10.14749-1400830395.

### Measures

The primary outcome variable HIV prevalence was based on HIV status divided into two categories: 1 = HIV positive individuals and 0 = HIV negative individuals. Explanatory variables included socio-demographic variables and HIV risk factors. All definitions of socio-demographic variables are based on Statistics South Africa [24]. The biological, socio-demographic and behavioural indicators are based on The Joint United Nations Programme on HIV and AIDS (UNAIDS) guidelines for second generation surveys [25]. The race categories used are consistent with those reported in the 2012 and previous national HIV prevalence, incidence and behaviour surveys. This is based on the question “Which of the following describes your population group? (Black African, White, Coloured, and Indian/Asian)”. The analysis was stratified by gender (males and females) and race, which was categorised into Black Africans and other (i.e., Whites, Coloureds, and Indian/Asian) due to the relatively smaller sample size for the latter race groups.

In the South African context, the historical apartheid regime defined concept of ‘race’ is still entrenched in society and population groups, and continue to be defined along a discrete racial classification namely Black African, White, Coloured and Indian [26, 27]. All South African citizens’ official identity books still carry their racial designation. These racial identities continue to be used in South Africa and have importance in cultural and social contexts as well as in the ongoing transformation process, with a view to addressing historical societal inequalities [2].

Other socio-demographic characteristics included age categories (15 to 24 years, 25 to 49 years, and 50+ years), marital status (married and not married), highest educational level attained (no education, primary, secondary, and tertiary), employment status (not employed and employed), locality type (urban formal, urban informal, rural informal, rural formal), and asset based socio-economic status (SES) constructed using multiple correspondence analyses (MCA) based on questions on availability of essential services and ownership of a range of household assets [28]. MCA is a data reduction technique for categorical data, which calculates a composite indicator score computed by adding up all weighted responses. The predicted score for each household was used to compute five quintiles representing a continuum of household SES from the most poor (lowest quintile) to the least poor (highest quintile). These five quintiles were categorised into low, middle, and high SES.

HIV-related risk factors included age at sexual debut (less than 15 years and more than 15 years), age disparate partnerships (partner older than 5 years, partner younger than 5 years, partner within 5 years), multiple sexual partners in the last 12 months (one partner, and two or more sexual partners), condom use at last sex (no and yes), alcohol use risk score (abstainers, low, high and hazardous risk drinkers) based on the Alcohol Use Disorder Identification Test (AUDIT) scale [29], self-perceived risk of HIV infection (no and yes), ever tested for HIV (no and yes), and awareness of HIV status (no and yes) based on the question “Have you ever tested for HIV, and if yes did you get your results back”.

### Statistical analysis

All statistical analyses were performed using Stata software version 12 (Stata Corp, College Station, Texas, USA) and the “svy” command to take into account complex survey design.

Descriptive statistics were used to characterize HIV prevalence by socio-demographic and HIV-related risk factors according to gender and race. Differences between categorical variables were assessed using the chi-square test. Bivariate logistic regression models were used to assess the relationship between HIV prevalence and each explanatory variable by gender and race. Statistically significant variables were entered into a multivariate logistic regression models to determine socio-demographic and HIV related risk factors associated gender and racial inequalities in HIV prevalence. Unadjusted odds ratios (OR), adjusted odds ratios (AOR) and their 95% confidence intervals (CI) with a *p*-value less than 0.05 are reported for all statistically significant results. Coefficient plots were used to display the results of the final multivariate model [30].

## Results

### HIV prevalence, gender and race groups

**Fig. 1:**
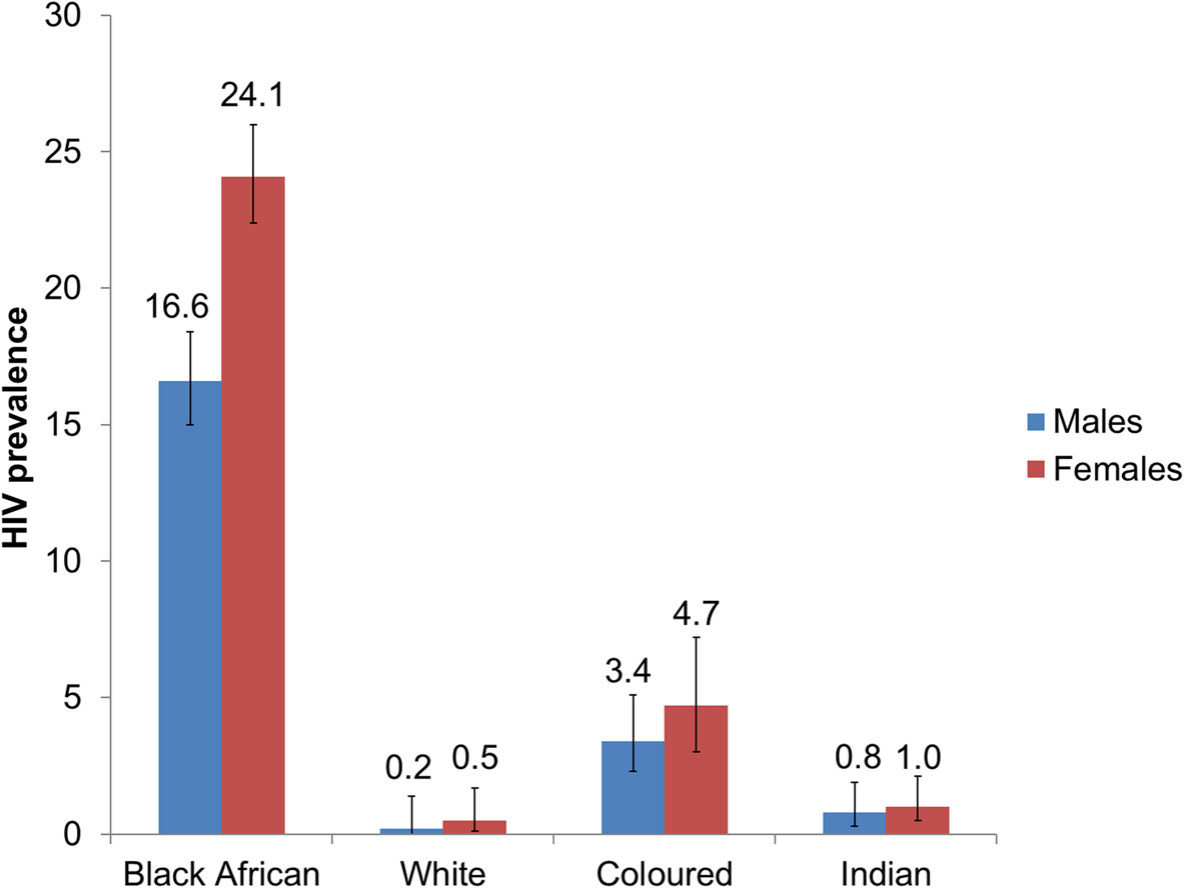
HIV prevalence with error bars among race groups by gender

Figure 1 shows racial and gender differences in HIV prevalence. Results suggest strong racial differences for HIV prevalence. HIV prevalence was significantly higher amongst Black Africans both males (16.6%; 95% CI: 15.0–18.4) and females (24.1%; 95% CI: 22.4–26.0) compared to their counterparts from other races (*p* < 0.001). Closer inspection of this trend showed that although there was a significant difference in HIV prevalence between male and female Black Africans, there was no significant gender difference in HIV prevalence for the other races (Fig. 1). For HIV prevalence, none of the other race groups differed significantly from each other. Hence, HIV prevalence for other race groups (White, Coloured, and Indian) was pooled in the subsequent analysis.

### HIV prevalence and socio-demographic characteristics

Table 1 shows the HIV prevalence stratified by socio-demographic characteristics, categorised race groups and gender. HIV prevalence was significantly higher among those with no education/primary education among both males (4.8%) and females (6.1%) of other race groups compared to those with higher educational attainment Furthermore, there was strong evidence that Black African females with secondary school education (27.2%) or lower education level (24.2%) had significantly higher HIV prevalence compared to those with tertiary education. Compared to their married counterparts, HIV prevalence was significantly higher among unmarried men of other races as well as all women.

**Table 1:** HIV prevalence and socio-demographic characteristics stratified by gender and race

Variables	Males								Females							
	Black African				Other races^a^				Black African				Other races^a^			
	n	HIV %	95% CI	*p*-values	n	HIV %	95% CI	*p*-value	n	HIV %	95% CI	*p*-value	n	HIV %	95% CI	*p*-value
Total	5202	16.6	15.0–18.4		3549	1.7	1.1–2.5		7348	24.1	22.4–26.0		4596	2.3	1.5–3.5	
Age (years)																
15 to 24	1940	3.3	2.5–4.5	< 0.001	854	0.6	0.3–1.5	< 0.001	2130	13.5	11.6–15.7	< 0.001	959	0.8	0.4–1.7	< 0.001
25 to 49	2120	25.7	22.8–28.8		1548	2.7	1.7–4.4		3173	36.0	33.0–39.2		1985	4.2	2.8–6.4	
50+	1140	12.2	9.4–15.7		1146	0.8	0.4–1.4		2043	10.1	8.5–12.1		1651	0.6	0.2–1.9	
Marital status																
Not Married	3773	17.2	15.2–19.4	0.248	1827	2.7	1.9–4.0	0.021	5512	26.9	24.8–29.1	< 0.001	2499	3.4	2.3–5.2	< 0.001
Married	1304	14.9	12.0–18.3		1676	0.9	0.4–2.3		1710	16.4	13.3–19.9		2046	0.7	0.3–1.7	
Education level																
No education/Primary	1148	17.4	14.4–20.9	0.078	439	4.8	2.8–8.1	< 0.001	1536	24.2	21.1–27.5	< 0.001	665	6.1	3.0–12.2	< 0.001
Secondary	2953	16.8	14.8–19.1		2392	1.5	0.9–2.5		4040	27.2	24.8–29.8		3043	2.2	1.4–3.5	
Tertiary	238	9.5	5.2–16.6		372	0.1	0.0–0.2		352	13.1	8.9–19.0		416	0.3	0.1–0.8	
Employment status																
Unemployed	2907	13.7	11.4–16.4	0.003	1207	2.5	1.5–4.2	< 0.001	5041	24.6	22.7–26.7	< 0.001	2623	3.7	2.3–5.9	< 0.001
Employed	1860	21.9	19.0–25.1		1930	1.6	0.9–2.8		1691	26.2	22.4–30.5		1540	1.2	0.7–2.0	
Asset based SES scores																
Low SES	3163	17.7	15.7–20.0	0.003	366	6.3	3.6–10.9	< 0.001	4327	27.3	25.1–29.6	< 0.001	395	8.0	4.6–13.4	< 0.001
Middle SES	1571	17.8	14.2–22.0		1215	3.6	2.0–6.4		2338	22.9	19.4–26.8		1710	4.3	2.5–7.3	
High SES	414	6.4	3.9–10.5		1922	0.5	0.3–1.0		590	9.6	6.8–13.6		2435	1.0	0.4–2.0	
Locality type																
Urban formal	1767	17	14.2–20.2	0.040	3016	1.5	1.0–2.4	< 0.001	2558	21.1	18.1–24.5	< 0.001	4010	2.3	1.5–3.7	< 0.001
urban informal	920	20.3	15.6–26.1		60	12.5	4.4–31.0		1192	34.4	30.1–39.0		79	11.5	6.7–19.1	
rural informal	1861	14.6	12.6–16.8		10	29.9	5.7–75.0		2987	24.3	22.0–26.9		5	22.7	4.3–66.1	
rural formal	654	23.8	16.7–32.8		463	1.3	0.6–3.0		611	31.3	26.9–36.2		502	1.6	0.8–3.2	

^a^Other races include White, Coloured and Indians/Asians

Generally, HIV prevalence was significantly higher among all men and women of low SES.

In addition, HIV prevalence was significantly higher among employed Black African males (21.9%) and females (26.2%) in contrast to other race groups whereby HIV prevalence was higher among unemployed males (2.5%) and females (3.7%) compared to those who were employed. Closer inspection of these trends shows a larger difference in the proportion of HIV infections among unemployed and employed Black African males (13.7% versus 21.9%) compared to the difference observed among women (24.6% versus 26.2%).

The HIV prevalence differed by locality type for Black African men and women. There was strong evidence that HIV prevalence was highest among Black African men (23.8%) living in rural formal areas in contrast with the highest overall HIV prevalence for Black African women living in urban informal areas (34.4%).

### HIV prevalence and behavioural risk factors

Table 2 shows the HIV prevalence and behavioural risk factors by gender and race group. HIV prevalence was significantly higher among those who reported having sexual partners younger than five years among Black African females (51.8%) than females compared with other age disparate partnerships. However, HIV prevalence was highest among women of other races who had sexual partners older than five years compared to other age disparate partnerships.

**Table 2:** HIV prevalence and HIV related risk factors stratified by gender and race

Variables	Males								Females							
	Black African				Other races^a^				Black African				Other races^a^			
	n	HIV %	95% CI	*p*-value	n	HIV %	95% CI	*p*-value	n	HIV %	95% CI	*p*-value	n	HIV %	95% CI	*p*-value
Total	5202	16.6	15.0–18.4		734	0.2	0.0–1.4		7348	24.1	22.4–26.0		873	0.5	0.1–1.7	
Age at sexual debut																
Younger than 15	393	14.3	10.2–19.7	0.096	26	5.1	0.7–29.8	0.489	229	26	18.3–35.6	0.801	11	0		0.034
15+ years	3318	18.9	17.0–21.1		568	0	0.0–0.1		5498	27.2	24.9–29.5		746	0.6	0.2–2.0	
Sexual partners in the last 12 month																
1 partner	2575	20.3	18.0–22.9	0.214	474	0.3	0.0–2.0	0.867	3822	28	25.3–30.8	0.210	546	0.8	0.2–2.7	0.311
2+ partner	663	16.9	12.9–21.8		23	0			211	34.5	25.3–45.0		17	0		
Age disparate partnerships																
5 years+ older	88	22.7	12.3–38.2	0.208	31	0.2	0.0–1.3	0.090	1694	29.4	25.6–33.5	0.001	165	1.6	0.3–7.4	0.035
5 years + younger	1163	21.4	18.1–25.2		132	1	0.2–6.4		98	51.8	38.1–65.3		26	0.4	0.1–3.0	
Within 5 years older or younger	1965	17.5	14.9–20.4		334	0			2239	26.3	23.4–29.4		373	0.3	0.0–2.3	
Condom use last sex act																
No	1806	16.2	13.6–19.1	0.002	411	0		0.002	2445	21.4	18.6–24.5	< 0.001	507	0.5	0.1–3.3	< 0.001
Yes	1363	23.4	20.1–27.1		68	1.9	0.3–11.4		1523	39.1	34.9–43.5		49	2.9	0.7–11.3	
Alcohol use risk score^b^																
Abstainers	2894	16.2	13.4–19.3	0.610	214	0.9	0.1–5.4	0.040	6070	24.2	22.3–26.1	0.768	402	1.1	0.3–3.7	< 0.001
Low risk drinkers (1–7)	1315	16	13.0–19.7		397	0	0.0–0.1		537	24.7	18.7–31.8		345	0	0.0–0.1	
High risk drinkers (8–19)	353	21	14.6–29.3		31	0			88	19.6	10.5–33.6		7	0		
Hazardous drinkers 20+)	22	14.8	4.0–41.9		0	0			3	0			1	0		
HIV risk perception																
No	1310	27.4	24.0–31.1	< 0.001	16	1.3	0.3–6.1	< 0.001	2275	39.1	35.5–42.8	< 0.001	14	8.4	1.3–39.7	< 0.001
Yes	3810	12.1	10.1–14.5		701	0.2	0.0–1.6		4927	16.7	14.8–18.7		844	0.4	0.1–1.7	
Ever test for HIV																
No	2376	11.8	9.1–15.2	< 0.001	232	0		0.044	2127	11.6	9.5–14.2	< 0.001	353	0.7	0.1–4.5	0.005
Yes	2767	20.3	18.0–22.7		488	0.3	0.1–2.1		5136	28.5	26.2–30.9		507	0.4	0.1–1.5	
Awareness of HIV status																
No	3259	16.4	14.1–19.1	0.846	533	0	0.0–0.1	0.165	3775	22.3	20.3–24.5	0.015	698	0.6	0.2–2.1	0.108
Yes	1838	16.8	14.3–19.6		177	0.8	0.1–5.5		3388	26	23.5–28.7		161	0		

^a^Other races include White, Coloured and Indians/Asians, ^b^Alcohol risk score based on a questionnaire for Alcohol Use Disorder Identification Test (AUDIT)

Generally, for all groups HIV prevalence was significantly higher among those who reported using a condom at last sex compared to those who reported not using a condom. In addition, HIV prevalence was significantly higher among those who perceived themselves as not being at risk of HIV, regardless of race or gender. Notably the HIV prevalence was highest among Black African women (39.1%) who perceived themselves not to be at risk of HIV. Among those who reported ever having an HIV test, the HIV prevalence was significantly higher for Black African males (20.3%) and Black African females (28.5%).

### Determinants of HIV prevalence by gender and race

The results of the bivariate analysis assessing the relationship between HIV prevalence and each explanatory variable by gender and race will be included as Additional files. Additional file 1 shows bivariate associon between HIV prevalence and socio-demographic characteristics by gender and race. Additional file 2 shows bivariate association between HIV prevalence and HIV related risk factors by gender and race. Only coefficient plots of variables that were significant and included in the multivariate model are described below.

### Male model

**Fig. 2:**
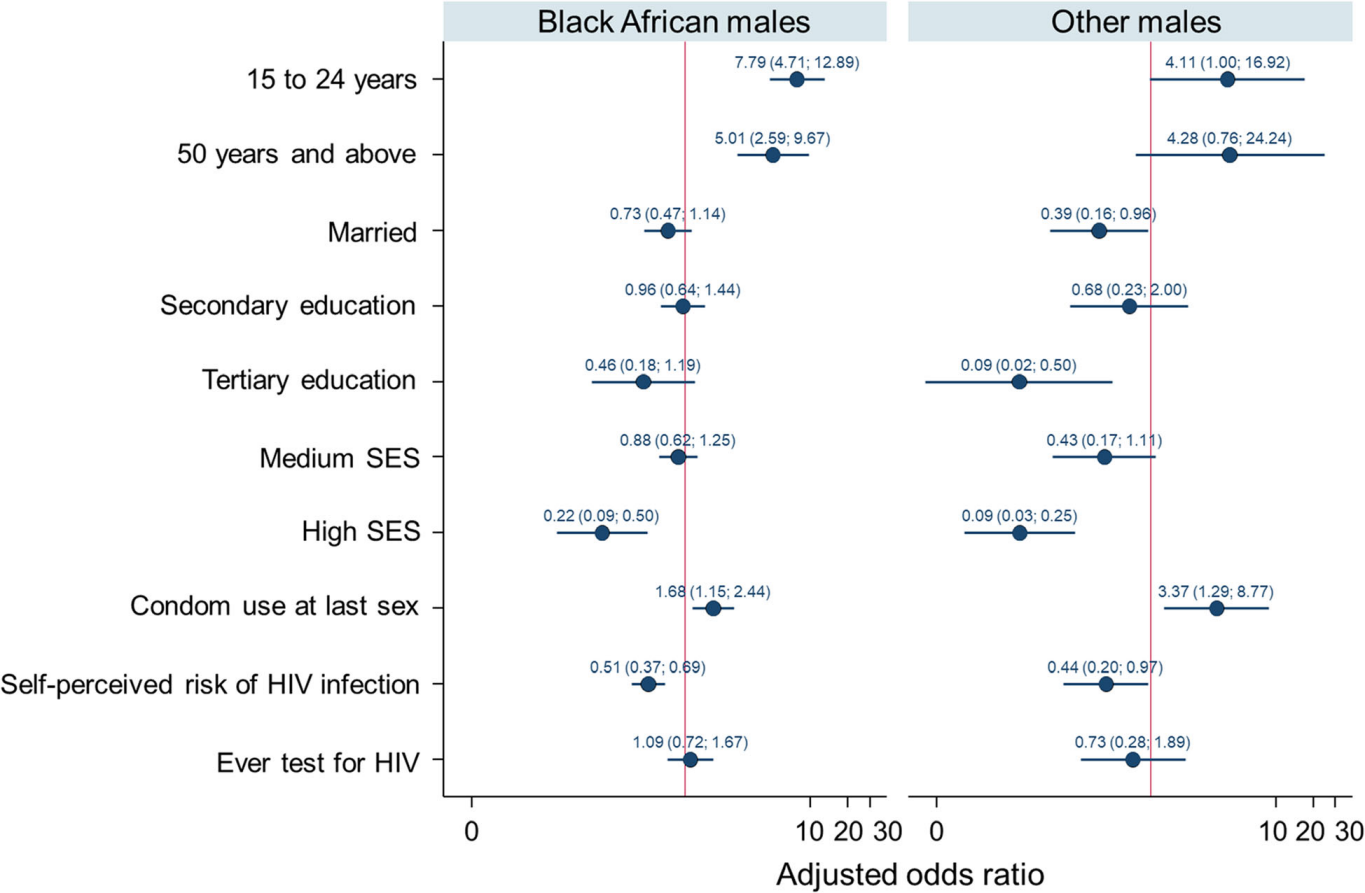
Multivariate logistic regression models of factors associated with HIV among Black African males and males from other race groups including White, Coloured and Indians/Asians

Figure 2 shows strong evidence of increased HIV prevalence among Black African males aged 25 to 49 years [AOR = 7.79 (95% CI: 4.71–12.89, p < 0.001] and those 50 years and older [AOR = 5.01 (95% CI: 2.59–9.67), p < 0.001] compared to the younger Black African males. A similar trend was observed among males of other races groups aged 25 to 49 years where there was evidence of high HIV prevalence risk compared to their younger counterparts [AOR = 4.11 (95% CI: 1.00 16.92), *p* = 0.051].

Reported condom use was significantly associated with increased prevalence of HIV infection among Black African males [AOR = 1.68 (95% CI: 1.15–2.44), *p* = 0.007] and males of other race groups [AOR = 3.37 (95% CI: 1.29–8.77), *p* = 0.013]. Generally, a high SES and self-perceived risk of contracting HIV was associated with a decreased risk of HIV. Among Black African males, a high SES [AOR = 0.22 (95% CI: 0.09–0.50) *p* < 0.001] and self-perceived risk of HIV [AOR = 0.51 (95% CI: 0.37–0.69), p < 0.001] was significantly associated with a decreased risk of HIV infection. The same trend was observed for males of other race groups, as high SES [AOR = 0.09 (95% CI: 0.03–0.25), *p* < 0.001] and self-perceived risk of HIV [AOR = 0.44 (95% CI: 0.20–0.97), *p* = 0.041] was associated with low risk of HIV.

### Female model

**Fig. 3:**
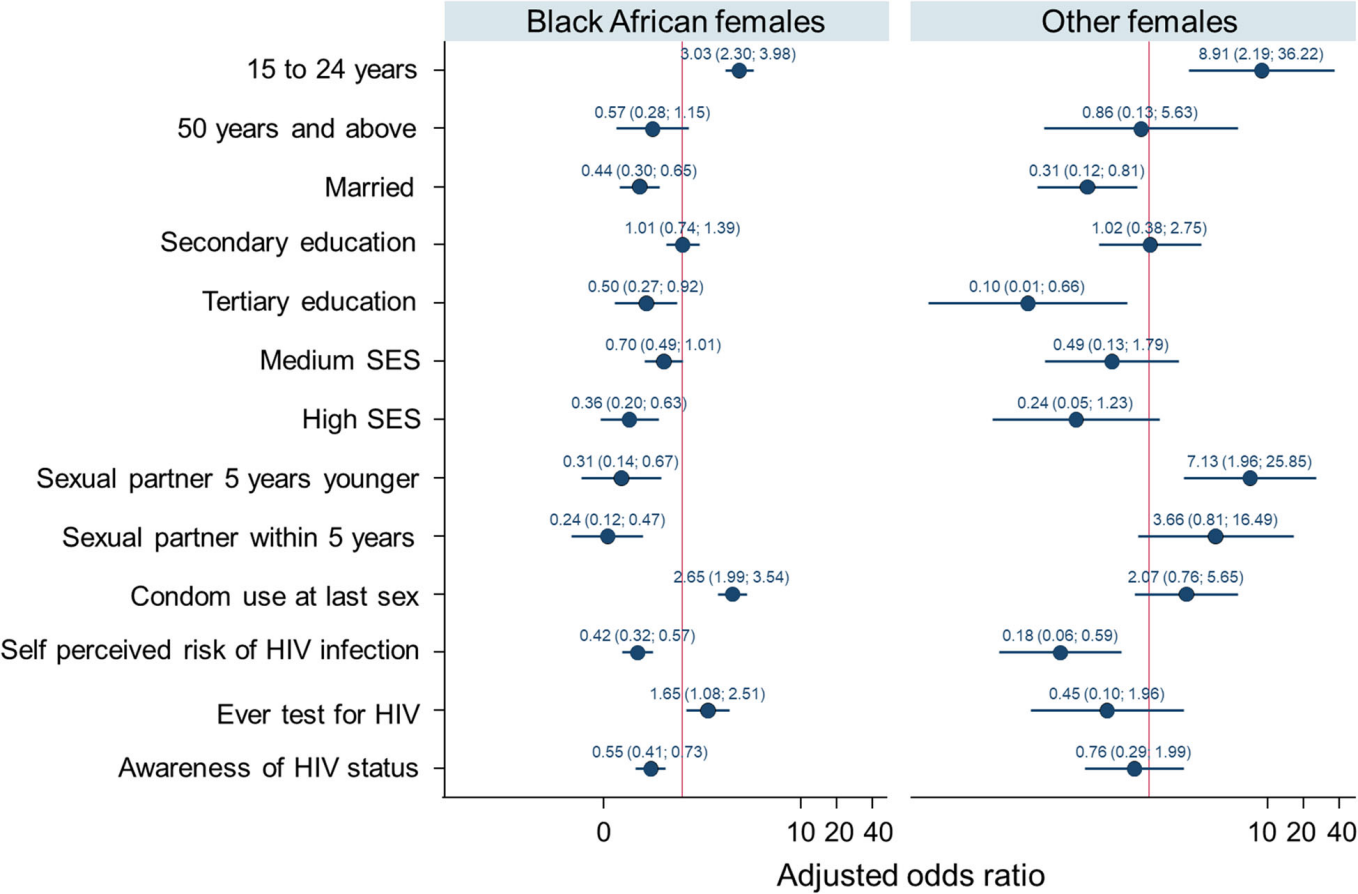
Multivariate logistic regression models of factors associated with HIV among Black African males and males from other race groups including White, Coloured and Indians/Asians

Figure 3 shows that among Black African females and females of other race groups increased prevalence of HIV infection remained significantly associated with age 25 to 49 years [OR = 3.03 (95% CI: 2.30–3.98), *p* < 0.001], and [OR = 8.91 (95% CI: 2.19–36.22), *p* = 0.002], respectively. Reported condom use at last sex and ever testing for HIV were significantly associated with increased prevalence of HIV infection only among Black African females with AOR = 2.65 (95% CI: 1.99–3.54), *p* < 0.001 and AOR = 1.65 (95% CI: 1.08–2.51), *p* = 0.020, respectively.

Lower prevalence of HIV infection was significantly associated with marriage [AOR = 0.44 (95% CI: 0.30–0.65), *p* < 0.001], tertiary education [AOR = 0.50 (95% CI: 0.27–0.92) *p* = 0.025], high SES [AOR = 0.36 (95% CI: 0.20–0.63) *p* < 0.001], partner 5 years younger [OR = 0.31 (95% CI: 0.14–0.67) *p* = 0.003], partner age difference within 5 years [AOR = 0.24 (95% CI: 0.12–0.47) *p* = 0.000], perceived risk of HIV infection [AOR = 0.42 (95% CI: 0.32–0.57) *p* < 0.001] and awareness of HIV status [AOR = 0.55 (0.41–0.73) *p* < 0.001] among Black African females. Similarly, among females of other race groups lower prevalence of HIV infection was significantly associated with marriage [AOR = 0.31 (95% CI: 0.12–0.81) *p* = 0.017), tertiary education [AOR = 0.10 (95% CI: 0.01 0.66), *p* = 0.018], partner age difference of less than five years [AOR = 0.14 (95% CI: 0.04–0.51) *p* = 0.003], and self-perceived risk of HIV infection [AOR = 0.45 (95% CI: 0.10–1.96) *p* = 0.005].

## Discussion

This study investigates factors linked with gender and racial disparities in HIV using the 2012 South African population-based national household survey. The findings revealed that some socio-demographic and behavioural factors related to HIV prevalence were common across the gender and racial divide but more prevalent among Black African females. Generally, HIV prevalence was higher among the productive and reproductive age groups, especially among those with no education or low educational qualifications and those residing in low and middle SES households. There were also factors that were unique to a particular gender and race group such as the higher prevalence of HIV among the employed Black Africans compared to the unemployed males in the other race groups. There was also a higher prevalence of HIV found in urban informal and rural formal areas among Black Africans compared to other race groups.

The current findings confirm that Black Africans in South Africa still carry the greatest burden of HIV. This coincides with low education levels, unemployment, and poverty as shown in other studies [31, 32]. These factors reflect the structural disparities which defined the fabric of South African society. The findings are in agreement with previous studies that have linked the heterogeneous HIV prevalence to the largely unequal socio-economic status of South Africans. The structural inequalities manifests itself in HIV related disparities along race and gender lines, with Black Africans being the most vulnerable [9, 33].

The lack of differences in behavioural factors associated with HIV prevalence between black African males and males of other race groups is contrary to the notion that Black African males suffer from greater HIV prevalence because they are considered less sexually responsible than their counterparts in other racial groups. Similarly, the findings also suggest that the high rates of HIV among Black African females is not just a simple result of high-risk behaviour but intrinsic structural inequalities that make them more likely to come into contact with the disease [21].

The current findings suggest that for women, being married and having a tertiary education are protective of HIV infection, regardless of race. In sub-Saharan Africa, associations between marriage and HIV infection among women vary and are generally complex and depend on both the type of relationship and sexual context [34]. The combined protective association with higher level of education could be indicative of the women’s social status, economic independence, and the ability to choose low risk sexual partners, thus limiting their risk of HIV infection [35]. Improving the educational and economic status of women has been shown to determine their bargaining power in sexual decision making even in marriage [36].

Findings also showed that for women having a sexual partner five years younger compared to having a sexual partner five years and older was protective of HIV infection, regardless of race. The influence of the interplay between age disparate relationships and gender-based power dynamics has long been shown to influence the risk of HIV infection [37–39]. Women with sexual partners at least five years older have been shown to be unable to negotiate safe sex for protection against HIV infection. This risk taking behaviour is influenced among other factors by socio-cultural dynamics amplified by gender norms, stereotypes and poverty, especially among Black Africans [37–39]. There is therefore a need to educate and empower women to self-protect against risky sexual behaviour irrespective of the age difference between themselves and their partners and socio-cultural norms and expectations.

The observed association between high SES and reduced HIV risk among all males, regardless of race is indicative of intrinsic socio-economic inequality that put males from low SES households at high risk of HIV infection. This is consistent with other work showing that groups that have been socially or economically marginalised are particularly vulnerable to HIV infection [40]. Despite the heightened risk of HIV among Black African males, HIV interventions should focus on the poorest without neglecting males in other race groups, as HIV risk is high for everyone in this stratum. However, there is still a need for better understanding of the nature and dynamics of the HIV epidemic in the disadvantaged minority race groups.

Males and females who reported condom use at last sex were at increased risk of HIV, regardless of race. This could be reflective of the fact that self-reported condom use at last sex might have not been a consistent self-reported practice hence the higher prevalence of HIV in this group. Another possible explanation for this could be social desirability bias in that the participant felt compelled to state they had used a condom. In addition, self-perceived risk of HIV was associated with a lower risk of HIV among both males and females, regardless of race. Furthermore, awareness of HIV status was associated with decreased risk of HIV infection among Black African women. These findings are positive attribute in the sense that HIV ignorance is no longer a major issue in the South African setting given more than 20 years of concerted efforts in the fight against the HIV epidemic. However, further research is needed to explore the link between awareness of HIV status, condom use at last sex and consistency of condom use and self-perceived risk of HIV with a gender and racial lens in South Africa.

## Limitations

The study’s cross-sectional design limits the ability to draw conclusions with regard to causality between the exposure and outcome variables. Furthermore, the survey data on sexual behaviour are based on self-reports. Hence, they are subject to both social desirability and recall bias. Unmeasured factors not adjusted for during analysis might contribute substantially to the difference in HIV/AIDS prevalence by gender and race. Despite these limitations, the study adds to existing literature and contributes to the understanding of the factors influencing gender and racial disparities in HIV in the South African context. Thus it has important implications for HIV risk prevention interventions within the gender and racial divide in the country. In addition, the survey data are based on a large nationally representative sample that can be generalized to the South African population.

## Conclusion

The findings reaffirm that gender and racial inequalities, as perpetuated by structural inequalities, such as educational attainment, socio-economic position, and contextual factors, such as socio-cultural norms, stereotypes, and beliefs, predisposes Black Africans and in particular women to heightened risk of HIV infection. Efforts to alleviate systemic societal inequalities based on gender and race should be viewed as part of a broader public health strategy to control and manage HIV as a chronic illness among the most marginalised groups. These groups have historically been disproportionately poor compared to their counterparts of other races. The gendered differences between men’s and women’s risk which cut across the different race groups reflect deep social differences in the cultural construction of gender roles [41]. This reinforces intrinsic gender based power dynamics, which compel women into relationships that expose them to increased risk of HIV infection. Given the high rates of unplanned pregnancies among young women in the country, this in turn may contribute to mother to child vertical transmission risk and hence perpetuating generational imbalances [42]. Therefore overcoming the scourge of HIV in Black African communities especially among women will take more than just biomedical interventions. The following actions are suggested:

- There is a need for continued effort to fix the fundamental societal and structural inequalities that are linked with the circumstances of HIV in the country
- There is a need to address the gendered dimension of the HIV epidemic closely related to socio-cultural patriarchal values, norms and stereotypes which marginalise women
- There is a need promote the education and empowerment of young girls and women for self-reliance and improved economic circumstances to break the cycles that underpin their vulnerabilities
- There is a need to develop suitable research frameworks for gender and race sensitive HIV data collection instruments that routinely document and monitor the impact of demographic, social, and economic conditions in order to inform HIV policies and programs

## Supplementary information

**Additional file 1.** Bivariate association between HIV prevalence and socio-demographic characteristics by gender and race.

**Additional file 2.** Bivariate association between HIV prevalence and HIV related risk factors by gender and race.

## Data Availability

The dataset(s) can be accessed upon request through the Human Sciences Research Council data research repository via http://www.hsrc.ac.za/en/research-data/.

## Abbreviations

AOR: Adjusted odds ratios
AUDIT: Alcohol Use Disorder Identification Test
CDC: Centre for Disease Control; confidence intervals
DBS: Dried blood spot
EAs: Enumeration areas
EIA: Enzyme immunoassay
HIV: Human immunodeficiency virus
MCA: Multiple Correspondence Analyses
NJ: New Jersey
OR: Odds ratios
SES: Socio-economic status
STD: Sexually Transmitted Diseases
TB: Tuberculosis
UNAIDS: The Joint United Nations Programme on HIV and AIDS
USA: United States of America
