# Supplementary material for: HIV prevalence in South Africa through gender and racial lenses: results from the 2012 population-based national household survey
Source: Int J Equity Health. 2019 Oct 30;18:167. doi: 10.1186/s12939-019-1055-6 (PMC6821038; doi:10.1186/s12939-019-1055-6)
Supplement: Supplementary file 1 — Additional file 1. Bivariate association between HIV prevalence and socio-demographic characteristics by gender and race. [file 12939_2019_1055_MOESM1_ESM.docx]

Additional file 1: Bivariate association between HIV prevalence and socio-demographic characteristics by gender and race

| **Variables** | **Males** | | | | | | | | **Females** | | | | | | | |
| --- | --- | --- | --- | --- | --- | --- | --- | --- | --- | --- | --- | --- | --- | --- | --- | --- |
|  | **Black African** | | | | **Other races*** | | | | **Black African** | | | | **Other races*** | | | |
| **Age (years)** | OR | 95% CI | | p-value | OR | 95% CI | | p-value | OR | 95% CI | | p-value | OR | 95% CI | | p-value |
| 15 to 24 | Ref |  |  |  | Ref |  |  |  | Ref |  |  |  | Ref |  |  |  |
| 25 to 49 | 10.0 | 6.9 | 14.5 | < 0.001 | 4.5 | 1.6 | 12.6 | 0.004 | 3.6 | 2.9 | 4.4 | < 0.001 | 5.2 | 2.3 | 11.5 | < 0.001 |
| 50+ | 4.0 | 2.6 | 6.3 | < 0.001 | 1.3 | 0.4 | 3.8 | 0.648 | 0.7 | 0.6 | 0.9 | 0.011 | 0.7 | 0.2 | 2.7 | 0.627 |
| **Marital status** |  |  |  |  |  |  |  |  |  |  |  |  |  |  |  |  |
| Not Married | Ref |  |  |  | Ref |  |  |  | Ref |  |  |  | Ref |  |  |  |
| Married | 0.8 | 0.6 | 1.1 | 0.248 | 0.3 | 0.1 | 0.9 | 0.027 | 0.5 | 0.4 | 0.7 | < 0.001 | 0.2 | 0.1 | 0.5 | < 0.001 |
| **Education level** |  |  |  |  |  |  |  |  |  |  |  |  |  |  |  |  |
| No education/Primary | Ref |  |  |  | Ref |  |  |  | Ref |  |  |  | Ref |  |  |  |
| Secondary | 1.0 | 0.7 | 1.3 | 0.778 | 0.3 | 0.1 | 0.6 | 0.002 | 1.2 | 1.0 | 1.4 | 0.128 | 0.3 | 0.2 | 0.8 | 0.010 |
| Tertiary | 0.5 | 0.3 | 1.0 | 0.042 | 0.0 | 0.0 | 0.0 | < 0.001 | 0.5 | 0.3 | 0.7 | 0.001 | 0.0 | 0.0 | 0.2 | < 0.001 |
| **Employment status** |  |  |  |  |  |  |  |  |  |  |  |  |  |  |  |  |
| Unemployed | Ref |  |  |  | Ref |  |  |  | Ref |  |  |  | Ref |  |  |  |
| Employed | 1.8 | 1.3 | 2.4 | < 0.001 | 0.6 | 0.3 | 1.4 | 0.240 | 1.1 | 0.9 | 1.4 | 0.455 | 0.3 | 0.2 | 0.6 | < 0.001 |
| **Asset based SES scores** |  |  |  |  |  |  |  |  |  |  |  |  |  |  |  |  |
| Low | Ref |  |  |  | Ref |  |  |  | Ref |  |  |  | Ref |  |  |  |
| Middle | 1.0 | 0.7 | 1.4 | 0.993 | 0.6 | 0.2 | 1.3 | 0.181 | 0.8 | 0.6 | 1.0 | 0.047 | 0.5 | 0.2 | 1.2 | 0.113 |
| High | 0.3 | 0.2 | 0.6 | < 0.001 | 0.1 | 0.0 | 0.2 | < 0.001 | 0.3 | 0.2 | 0.4 | < 0.001 | 0.1 | 0.0 | 0.3 | < 0.001 |
| **Locality type** |  |  |  |  |  |  |  |  |  |  |  |  |  |  |  |  |
| Urban formal | Ref |  |  |  | Ref |  |  |  | Ref |  |  |  | Ref |  |  |  |
| urban informal | 1.2 | 0.8 | 1.8 | 0.260 | 9.3 | 2.7 | 31.8 | < 0.001 | 2.0 | 1.5 | 2.6 | < 0.001 | 5.5 | 2.6 | 11.8 | < 0.001 |
| rural informal | 0.8 | 0.6 | 1.1 | 0.191 | 27.6 | 3.6 | 214.3 | 0.002 | 1.2 | 0.9 | 1.5 | 0.127 | 12.4 | 1.7 | 88.7 | 0.012 |
| rural formal | 1.5 | 0.9 | 2.5 | 0.091 | 0.9 | 0.3 | 2.3 | 0.787 | 1.7 | 1.3 | 2.3 | < 0.001 | 0.7 | 0.3 | 1.6 | 0.397 |

*Other races include White, Coloured, and Indians/Asians
