# Supplementary material for: HIV prevalence in South Africa through gender and racial lenses: results from the 2012 population-based national household survey
Source: Int J Equity Health. 2019 Oct 30;18:167. doi: 10.1186/s12939-019-1055-6 (PMC6821038; doi:10.1186/s12939-019-1055-6)
Supplement: Supplementary file 2 — Additional file 2. Bivariate association between HIV prevalence and HIV related risk factors by gender and race. [file 12939_2019_1055_MOESM2_ESM.docx]

Additional file 2: Bivariate association between HIV prevalence and HIV related risk factors by gender and race

| **Variables** | **Males** | | | | | | | | **Females** | | | | | | | |
| --- | --- | --- | --- | --- | --- | --- | --- | --- | --- | --- | --- | --- | --- | --- | --- | --- |
|  | **Black African** | | | | **Other races*** | | | | **Black African** | | | | **Other races*** | | | |
| **Age at sexual debut** | OR | 95% CI | | p-value | OR | 95% CI | | p-value | OR | 95% CI | | p-value | OR | 95% CI | | p-value |
| Younger than 15 | Ref |  |  |  | Ref |  |  |  | Ref |  |  |  | Ref |  |  |  |
| 15+ years | 1.4 | 0.9 | 2.1 | 0.097 | 0.6 | 0.1 | 2.6 | 0.494 | 1.1 | 0.7 | 1.7 | 0.801 | 0.3 | 0.1 | 1.0 | 0.043 |
| **Sexual partners in the last 12 months** |  |  |  |  |  |  |  |  |  |  |  |  |  |  |  |  |
| 1 partner | Ref |  |  |  | Ref |  |  |  | Ref |  |  |  | Ref |  |  |  |
| 2+ partn | 0.8 | 0.6 | 1.1 | 0.215 | 0.9 | 0.3 | 2.4 | 0.867 | 1.4 | 0.8 | 2.2 | 0.212 | 2.3 | 0.4 | 12.5 | 0.325 |
| **Age disparate partnerships** |  |  |  |  |  |  |  |  |  |  |  |  |  |  |  |  |
| 5 years+ older | Ref |  |  |  | Ref |  |  |  | Ref |  |  |  | Ref |  |  |  |
| 5 years + younger | 0.9 | 0.4 | 2.0 | 0.854 | 2.2 | 0.5 | 10.0 | 0.293 | 2.6 | 1.4 | 4.6 | 0.001 | 0.3 | 0.1 | 0.9 | 0.039 |
| Within 5 years older or younger | 0.7 | 0.3 | 1.6 | 0.414 | 1.0 | 0.2 | 4.4 | 0.966 | 0.9 | 0.7 | 1.1 | 0.175 | 0.4 | 0.1 | 1.0 | 0.046 |
| **Condom use last sex act** |  |  |  |  |  |  |  |  |  |  |  |  |  |  |  |  |
| No | Ref |  |  |  | Ref |  |  |  | Ref |  |  |  | Ref |  |  |  |
| Yes | 1.6 | 1.2 | 2.1 | 0.002 | 3.2 | 1.5 | 7.0 | 0.004 | 2.4 | 1.8 | 3.0 | <0.001 | 6.5 | 2.4 | 17.6 | <0.001 |
| **AUDIT**** |  |  |  |  |  |  |  |  |  |  |  |  |  |  |  |  |
| Abstainers | Ref |  |  |  | Ref |  |  |  | Ref |  |  |  | Ref |  |  |  |
| Low risk drinkers (1-7) | 1.0 | 0.7 | 1.4 | 0.967 | 1.6 | 0.6 | 3.9 | 0.321 | 1.0 | 0.7 | 1.5 | 0.878 | 0.8 | 0.4 | 1.5 | 0.480 |
| High risk drinkers (8-19) | 1.4 | 0.8 | 2.4 | 0.245 | 3.8 | 1.4 | 10.4 | 0.010 | 0.8 | 0.4 | 1.6 | 0.475 | 12.2 | 3.1 | 48.5 | <0.001 |
| Hazardous drinkers (20+) | 0.9 | 0.2 | 3.9 | 0.888 | 4.7 | 0.7 | 31.0 | 0.110 | 1.0 |  |  |  | 19.0 | 2.3 | 158.2 | 0.007 |
| **Risk of HIV Infection** |  |  |  |  |  |  |  |  |  |  |  |  |  |  |  |  |
| No | Ref |  |  |  | Ref |  |  |  | Ref |  |  |  | Ref |  |  |  |
| Yes | 0.4 | 0.3 | 0.5 | <0.001 | 0.2 | 0.1 | 0.4 | <0.001 | 0.3 | 0.3 | 0.4 | <0.001 | 0.1 | 0.0 | 0.1 | <0.001 |
| **Level of risk perception** |  |  |  |  |  |  |  |  |  |  |  |  |  |  |  |  |
| Low risk | Ref |  |  |  | Ref |  |  |  | Ref |  |  |  | Ref |  |  |  |
| High risk | 2.7 | 2.1 | 3.6 | <0.001 | 5.1 | 2.6 | 9.8 | <0.001 | 3.2 | 2.6 | 3.9 | <0.001 | 16.0 | 7.1 | 36.3 | <0.001 |
| **Ever test for HIV** |  |  |  |  |  |  |  |  |  |  |  |  |  |  |  |  |
| No | Ref |  |  |  | Ref |  |  |  | Ref |  |  |  | Ref |  |  |  |
| Yes | 1.9 | 1.4 | 2.7 | <0.001 | 2.0 | 1.0 | 4.0 | 0.048 | 3.0 | 2.4 | 3.9 | 0.000 | 4.0 | 1.4 | 11.5 | 0.009 |
| **Awareness of HIV status** |  |  |  |  |  |  |  |  |  |  |  |  |  |  |  |  |
| No | Ref |  |  |  | Ref |  |  |  | Ref |  |  |  | Ref |  |  |  |
| Yes | 1.0 | 0.8 | 1.4 | 0.845 | 1.7 | 0.8 | 3.6 | 0.170 | 1.2 | 1.0 | 1.4 | 0.015 | 1.8 | 0.9 | 3.7 | 0.113 |

*Other races include White, Coloured, and Indians/Asians, **Alcohol risk score based on a questionnaire for Alcohol Use Disorder Identification Test (AUDIT)
